# Audit of imaging documentation on an ICU

**DOI:** 10.1186/cc14595

**Published:** 2015-03-16

**Authors:** N Arora, SJ Glover

**Affiliations:** 1 Good Hope Hospital, Sutton Coldfield, UK

## Introduction

The Ionising Radiation (Medical Exposure) Regulations 2001 recommend to 'ensure that a clinical evaluation of the outcome of each medical exposure is recorded' [1]. This audit looked at whether ICU documentation of investigations involving ionising radiation could be improved. Anticipated benefits would be improved communication between the multidisciplinary team and better-informed decision-making.

## Methods

Patients admitted to the ICU between 21 September 2014 and 2 October 2014 were included. If an investigation did not involve ionising radiation or was not requested by intensive care clinicians it was excluded. The indication for imaging was noted, and patient notes were analysed no less than 48 hours after the imaging was reported.

## Results

**Figure 1:**
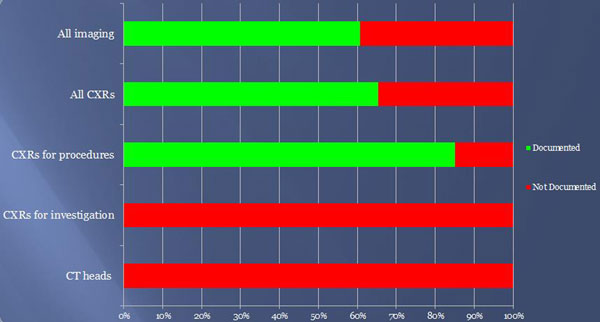


As shown in Figure 1, imaging requests were generally poorly documented (61%). In total, 17/26 (65%) chest X-rays (CXRs) were documented. A total of 0/2 CT scans were documented, despite one showing acute changes. In total, 17/20 (85%) CXRs requested following procedures carried out on ITU (such as insertion of central venous catheters) were documented, and the three not documented had no significant findings. The six other CXRs were requested to investigate worsening respiratory function. None were documented. Five had significant findings.

## Conclusion

Investigations following procedures were generally well documented, but investigations seeking pathology were not documented at all, regardless of the findings. This may have influenced the management of the patient and compromised patient safety. As such, the audit was presented at a departmental meeting to emphasise the importance of imaging documentation. A place for investigations was added to the ICU patient list to improve communication between the team, and a second audit is planned to assess the impact of this.

## References

[B1] The ionising radiation (medical exposure) regulations 2000 2000 London: Stationery Office

